# Green synthesis of cubic CuO nanoparticles for biomedical applications and the photodegradation of methylene blue: RSM-BBD optimization of the reaction parameters and stability studies

**DOI:** 10.1039/d5na01159k

**Published:** 2026-04-28

**Authors:** Abu Bakar Siddique, Azhar Abbas, Muhammad Sher, Yasir Zaman, Muhammad Fayyaz ur Rehman, Umar Nishan, Ibrahim A. Shaaban

**Affiliations:** a Institute of Chemistry, University of Sargodha Sargodha 40100 Pakistan abubakar.siddique@uos.edu.pk; b Department of Chemistry, Government Ambala Muslim College Sargodha 40100 Pakistan; c Department of Physics, University of Sargodha Sargodha 40100 Pakistan; d Department of Chemistry, Kohat University of Science and Technology Kohat 26000 KP Pakistan; e Department of Chemistry, Faculty of Science, Research Center for Advanced Materials Science (RCAMS), King Khalid University P.O. Box 960 Abha 61421 Saudi Arabia

## Abstract

Aquatic pollution poses an immense risk to human health and environmental preservation, with azo dyes from textile wastewater being a major source. Nanotechnology offers excellent methods for wastewater treatment, particularly through sunlight-driven photocatalysis. In this study, copper oxide nanoparticles (CMFE@CuO NPs) were produced *via* an eco-friendly green approach using the *C. macrocarpa* fruit extract. The produced NPs were thoroughly examined using advanced analytical techniques, revealing a crystallite size of 16.9 nm and high stability. The CMFE@CuO NPs displayed outstanding photocatalytic performance for methylene blue degradation under sunshine. The reaction conditions were tuned *via* the response surface approach based on a Box–Behnken design. Under optimal parameters (50 mg catalyst, 10 ppm dye, pH 8, and temperature 358 K), 99.9% dye degradation was achieved within 120 minutes, at a rate constant of 4.44 × 10^−2^ min^−1^. Total organic carbon analysis demonstrated 81% mineralization within 4 hours, while regeneration experiments confirmed significant reusability with only a 7% activity decrease after five cycles. Radical scavenging investigations supported the postulated degradation mechanism. In addition to photocatalytic activity, the CMFE@CuO NPs demonstrated considerable biological performance, demonstrating strong antibacterial efficacy and high antioxidant activity, comparable to gallic acid. Overall, the findings indicate CMFE@CuO NPs as very effective, reusable, and versatile nanocatalysts for wastewater treatment and environmental remediation.

## Abstract 2

Green synthesis of CMFE@CuO nanoparticles and evaluation of their biological and photocatalytic applications for water treatment.

## Graphical abstract

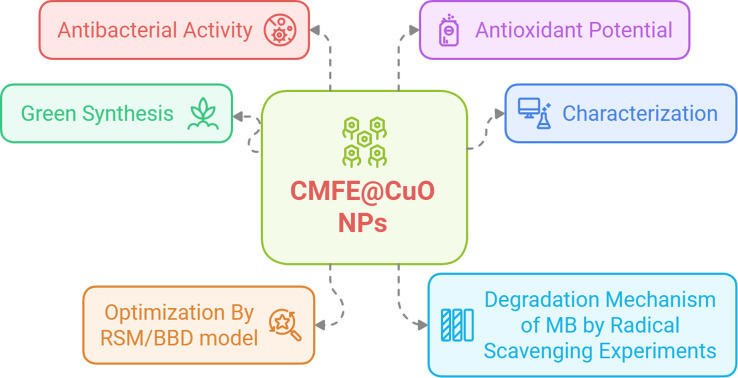

## 1. Introduction

Our environment has suffered an immense transformation in recent years due to advances in industry. Industrial effluents are the primary cause of environmental pollution, and these wastes continuously poison water bodies.^1^ Water bodies are contaminated by a variety of organic and inorganic pollutants, heavy metals, and endocrine-disrupting chemicals discharged from food, pharmaceutical, leather, textile, and cosmetic facilities. Among these pollutants, azo dyes exhibit the most radical effects in disrupting the aquatic ecosystem.^2^ Over 70% of 900 000 metric tons of dyes produced each year are azo dyes.^3^ These dyes present in the effluents are directly mixed with freshwater reservoirs, resulting in the disruption of the photosynthetic activity of aquatic plants by limiting the sunlight and oxygen demand.^4^ Among various hazardous dyes, methylene blue (MB), a cationic azo dye, is known to cause numerous ailments, including skin and eye irritation, gastric disorders, respiratory issues, and even cancer. MB is also reported to cause hypertension, precordial pain, fever, headache, and bladder irritation.^5^ Therefore, the mitigation or complete removal of these pollutants is necessary for a balanced aquatic ecosystem and ultimately for human health.^6,7^ MB is a popular model dye in photodegradation studies due to its vivid coloration, distinct UV-visible spectral peaks, extensive use in the textile industry, and accessibility, making it a relevant subject for wastewater treatment research.^8^

Among many methods, such as adsorption, chemical oxidation, precipitation, and photocatalytic degradation, applied for organic pollutant removal, photodegradation is considered the most viable, economic, and environmentally benign approach.^9^ Photodegradation using nanomaterials requires neither hazardous chemicals nor high energy and results in the complete oxidation of azo dyes into carbon dioxide and water. Upon exposure to suitable light wavelengths, metal-based nanoparticles (NPs) are activated to generate electron–hole (e^−^/h^+^) pairs, actively involved in the production of reactive oxygen species (ROS). These ROS can easily destroy the covalent bonds of azo dyes by redox reactions and convert them into less or non-hazardous species.^10^ Many metal-based NPs have been explored previously for photodegradation potential, but these materials still face challenges in achieving the complete mineralization of azo dyes in the shortest time.^10,11^ In this regard, greenly synthesized NPs are highly preferred due to their easy generation, non-hazardous nature, large surface area, and efficient sunlight response to degrade pollutants.

Sunlight-responsive copper oxide NPs (CuO NPs) are important materials that can effectively degrade organic pollutants under sunlight within a short time. Due to their large volume-to-surface area ratio, sunlight-responsive bandgap, high stability at high temperature, and cost-effective raw materials, CuO NPs are believed to be important candidates as photocatalysts.^12^ The optical and catalytic properties of CuO NPs can further be improved by surface functionalization with bioactives that can easily adsorb dye molecules on their surface.^13^ Several previous studies have shown the high catalytic and biological efficacy of greenly synthesized CuO NPs. For example, Atri *et al.* reported *Ephedra alata* extract-assisted biosynthesis of CuO NPs for the photodegradation of MB,^14^ and Koteeswari *et al.* documented the photodegradation of MB using CuO NPs produced *via* a papaya and banana peel-mediated synthesis.^12^ Additionally, in recent years, many CuO-based nanocomposites have attracted significant attention owing to their multifaceted applications in the field of catalysis and biology. For instance, Arulkumar *et al.* reported that CuO@Fe_2_O_3_ nanocomposites degraded crystal violet dye (92.82%) and exhibited antimicrobial activity against the growth of *S. aureus*, *E. coli* and *C. albicans*^15^. However, the quest for surface-modified, eco-friendly CuO NPs *via* green synthesis to completely degrade azo dyes through the facile generation of ROS is still ongoing.

Recently, numerous plant-based materials have been exploited for the eco-friendly synthesis of NPs, but the exploration of fruit extracts that are not widely used as food has emerged as an interesting area. Owing to their enriched phytochemical content, fruit extracts act as better bio-based reducing agents, with additional advantages including their natural and renewable origin, rich phytochemical composition, and non-toxic nature. Thus, they are environmentally friendly and sustainable compared with the toxic solvents or chemicals used in the chemical synthesis of NPs.^16,17^ Although the reduction efficiency of fruit extracts may vary depending on the nature, method of extraction and phytochemical composition, the use of these extracts still elicits better or at least a comparable reduction efficiency compared to other alternatives, like microbial or leaf extracts.^16,18,19^ In this regard, one of the phytochemically enriched fruits is the *C. macrocarpa* fruit, known as the natal plum. This fruit is a rich source of several antioxidants, vitamins, flavonoids, and phenolics.^20,21^ The aqueous extract of this fruit has been reported to synthesize many stable metal-based NPs, like AgNPs,^22^ AuNPs,^23^ CdO NPs,^24^ NiO NPs,^25^ and ZnO-NiO NCs,^26^ but it has never been explored for the synthesis of CuO NPs. Since no previous study has reported the synthesis of CuO NPs using *C. macrocarpa*, this research work might provide an essential addition to the existing literature on the cost-effective photodegradation of azo dyes and biomedical applications.

A number of reaction variables, *i.e.*, the pH of the dye solution, temperature, and dye concentration, also play important roles in the degradation efficiency of the catalyst. A high degradation efficiency by the catalyst can be achieved by optimizing the reaction conditions. A combination of experimental work and statistical calculations using machine learning tools is an advanced method for optimizing reaction parameters. The response surface methodology (RSM)-based optimization of reaction parameters has been extensively studied.^27^ Therefore, this method has been selected for the optimization of the photodegradation reaction, as evidenced by previously reported work.^28^ By optimizing the parameters, a high catalytic efficiency can be obtained, which is necessary for commercial applications.

In addition to catalytic applications, nanotechnology has also served humanity in tackling various biomedical issues, ranging from the eradication of microbial pollution and sensing of antibiotics to tissue generation and cancer therapy.^29–33^ Among several water pollution issues, microbial water pollution is an imminent threat to freshwater reservoirs.^34^ The exceptional antimicrobial and antioxidant properties of plant extracts further emphasize the potential of bio-fabricated NPs as potent antibacterial materials and antioxidants.^35^ These NPs can hinder the normal functioning of bacterial cells by easily crossing the membrane barriers due to their small size and phytochemically enriched surface. On entering bacterial cells, they interact with cellular organelles and the genome either directly or by producing ROS, causing various cell mutations that ultimately lead to bacterial death. Moreover, owing to the high reducing properties of CuO NPs and their phyto-functionalized surface, the greenly synthesized NPs may also reduce the oxidizing species produced as a result of various metabolic reactions. Therefore, these NPs can also have applications in creams, ointments, and surface cleanings.^36^

The current work presents the *C. macrocarpa* aqueous extract-based green synthesis of CuO NPs. The reported CuO NPs were thoroughly characterized *via* UV-visible and FTIR spectroscopy, PXRD, DLS and zeta potential, SEM, EDX, TGA, and TEM analyses. The post-characterized, phyto-functionalized CuO NPs were assessed for the photocatalytic disintegration of MB dye under intense sunlight, and the RSM-BBD model was adopted to optimize the reaction conditions. Afterward, the antibacterial and antioxidant potential of the CuO NPs was assessed for biological applications.

## 2. Experimental work

### 2.1. Chemicals and instruments used

AR-grade chemicals were procured from reliable suppliers, Sigma-Aldrich and Merck (Germany), to perform experiments. Ultra-pure deionized water was utilized as an aqueous medium for the preparation and dilution of all necessary solutions. Throughout the experimental work, the glassware was carefully cleaned with chromic acid, rinsed with distilled water and dried in a hot-air oven to ensure sterility. Fresh fruits of *C. macrocarpa* (Fig. S1) were obtained from the botanical sanctuary of the University of Sargodha, Pakistan.

The PXRD spectrum was recorded using the JDX-3532 diffractometer (JEOL, Japan), utilizing Cu-Kα radiation (*λ* = 1.5418 Å) in the 2*θ* range of 10–80°. FTIR analysis was conducted using an FTIR spectrophotometer (Shimadzu FTIR-8400S, Japan), while a UV-vis spectrophotometer (Shimadzu Pharmaspec-1700, Japan) was used to record the optical absorption spectra. The morphological analysis of the samples was performed using SEM (JSM5910, JEOL, Japan) and TEM (JEM-ARM2000F, JEOL, Japan).

### 2.2. Preparation of *C. Macrocarpa* fruit extract

The preparation of CMFE was carried out according to a methodology outlined in the literature.^21,37^ The initial steps involved in the aqueous extraction of *C. macrocarpa* fruits included washing with DW to remove surface contamination, shade-drying at room temperature, chopping to separate the seeds from the fruit pulp, and grinding of dried pulp to obtain a homogeneous powder. Subsequently, an accurately weighed amount (∼10 g) of the dried fruit powder was added to 100 mL of deionized water in a 250 mL round-bottom flask. The resulting solution was subjected to reflux at 90 °C and left to stand for 3 h while being magnetically stirred at 400 rpm. A deep red-coloured solution was obtained, cooled to room temperature and filtered to separate a bioactive-rich liquid extract from the solid residues. The clear extract was then evaporated in an oven at 80 °C for 24 h, and the subsequent dried powder (CMFE) was stored at 4 °C for later use in the bio-fabrication of NPs.

### 2.3. Phytochemical screening of CMFE

The rich presence of numerous bioactive entities, such as phenolics, carbohydrates, tannins, flavonoids, terpenoids, saponins, anthraquinone, and alkaloids, in CMFE was verified through a series of distinctive qualitative assays, namely the Lead Acetate, Molisch, Braymer, Shinoda, Salkowski, Foam, Borntrager, and Dragendorff tests, respectively.^21,38,39^

### 2.4. Biogenic preparation of CuO NPs

A typical green synthesis approach was employed to prepare CMFE@CuO NPs. To do this, the CMFE extract (20 mg, 15 mL) was added dropwise to 20 mL of a 30 mM Cu(NO_3_)_2_·2H_2_O solution under constant reflux at 100 °C, at a stirring rate of 200 rpm. To the above solution, a 0.1 M solution of NaOH was introduced dropwise to adjust its pH to 9, providing an alkaline medium for the production of stable NPs with a controlled size and defined morphology. Upon the appearance of brown precipitates in the mixture, the reaction was stopped, and the solution was cooled to room temperature. The obtained suspension was centrifuged at 8000 rpm to isolate the precipitates, which were then washed thrice with deionized water to remove the soluble salts and unused extract. The purified precipitates were then dried in an oven at 150 °C for 2 h and calcined in a muffle furnace at 400 °C for 3 h, yielding a uniform powder of CMFE@CuO NPs for further analysis.

### 2.5. Photocatalytic activity evaluation of CMFE@CuO NPs

The photocatalytic performance of freshly synthesized CMFE@CuO NPs was evaluated through the sunlight-driven degradation of methylene blue (MB) following a standard procedure.^40^ Briefly, CMFE@CuO NPs (10 mg) were dispersed in 20 mL of a 10 ppm MB solution and magnetically stirred in the dark at 200 rpm for 20 minutes to establish adsorption–desorption equilibrium. The reaction mixture was then exposed to direct sunlight for 2 hours in June (15–25 June, 2025), between 10:00 am and 02:00 pm at the University of Sargodha with an average light intensity of 900 W m^−2^. After every 15 minutes, aliquots were taken, and absorbance was measured to monitor the gradual decrease in MB absorbance at 668 nm. The photocatalytic degradation efficacy (%) was calculated using the absorbance data (eqn (1)), enabling quantitative assessment of the catalyst's performance as follows:^41,42^1
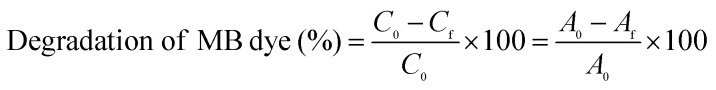


*C*_0_ and *C*_f_ denote the starting and final concentrations of the dye, respectively. Fig. S2 illustrates the overall framework of the photocatalytic activity investigations. Based on a comprehensive literature review, the impact of various reaction parameters, such as pH (3–13), catalyst dosage (10–50 mg), initial dye concentration (10–30 ppm), and temperature (298–358 K), on the degradation efficiency was also recorded.^23,43,44^ The statistical tool, *i.e.*, RSM/BBD, was employed to optimize these reaction variables.

### 2.6. Experimental design of RSM/BBD

RSM encompasses statistical and mathematical approaches employed to assess the interrelationships among various process variables affecting pollutant degradation.^28,45^ This work utilized the Box–Behnken design (BBD) to systematically optimize the experimental settings. Each variable was analyzed at two coded levels: low (−1) and high (+1), as detailed in Table S1. A total of 29 experimental runs were performed to analyze methylene blue degradation across the four specified parameters. The experimental results were later compared with model-predicted values to validate the reliability and appropriateness of the BBD model.

### 2.7. Total organic content (TOC) analysis

The fading of the dye solution's color does not reflect the complete mineralization of the dye molecules. The partial breakdown of dyes can lead to the creation of numerous harmful chemicals in water, which further impair the water quality.^42^ Dissociation of the diazenyl group induces substantial discoloration of the dye solution.^46^ Therefore, evaluating the total organic content (TOC) value is vital to verifying the complete mineralization of dyes. The TOC removal percentage was calculated using eqn (2) given below:^47^2
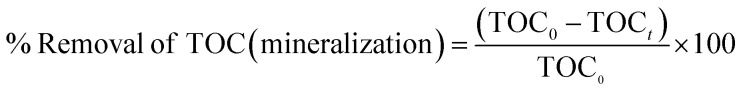
where TOC_0_ is the initial concentration of organic content, while TOC_*t*_ is the concentration of organic content after time ‘*t*’.

### 2.8. Catalyst reusability studies

To check the activity of the catalyst for repeated usage, reusability experiments were executed on the surface of CMFE@CuO NPs for five consecutive cycles. After each usage, CuO NPs were recovered using an already documented procedure.^48^ Soon after MB degradation, the catalyst was recovered by centrifugation of the resulting mixture for 30 min, rinsed with distilled water and reactivated by oven-drying for 3 hours. Afterward, the recovered catalyst was successfully employed for the next photocatalytic batch experiment.

### 2.9. Disc diffusion assay

The antibacterial activity of CMFE and the CMFE@CuO NPs was evaluated using the standard disc diffusion assay, following previously reported methods.^21,49^ Two Gram-negative bacteria (*Escherichia coli* and *Pseudomonas aeruginosa*) and two Gram-positive bacteria (*Staphylococcus aureus* and *Bacillus subtilis*) were selected to assess the bactericidal efficacy of the materials. Bacterial cultures were grown in nutrient agar (2.5 g/100 mL), and the cell density was adjusted to approximately 10^8^ CFU per mL with saline. Fresh agar medium (92.5 g/100 mL) was prepared, poured into Petri dishes, sterilized, and inoculated with the respective bacterial strains. After solidifying, sterile paper discs saturated with CMFE (2 mg/5 mL) and CMFE@CuO NPs (2 mg/5 mL) were placed on the agar surface. The plates were then left to incubate at 37 °C for 24 hours. The antibacterial activity was assessed by measuring the diameter of the inhibition zones created around each disc, and the results were analyzed to evaluate the relative efficacy of the materials against the tested bacterial strains.

The MIC values of CMFE and the CMFE@CuO NPs were also determined by the broth microdilution method for accurate quantification of the antibacterial activity. For this purpose, a series of aqueous dilutions of CMFE and CMFE@CuO NPs were prepared in the range of 50–500 µg mL^−1^, and fresh bacterial cultures of *E. coli*,*P. aeruginosa*,*S. aureus*, and *B. subtilis* were inoculated in nutrient broth and incubated at 37 °C overnight. After adding the CMFE and CMFE@CuO NP dilutions to the bacterial inoculum, they were subjected to overnight incubation at 37 °C. The MIC value against each bacterial strain was calculated and reported in µg mL^−1^.

### 2.10. DPPH assay

The antioxidant efficacy of the samples was tested using the frequently used DPPH radical scavenging assay, which is based on hydrogen atom transfer processes.^50^ A 0.1 mM DPPH solution (3.9 mg in 100 mL of methanol) was produced and stored in the dark for 2 hours. A blank was made by combining 1.2 mL of the DPPH solution with 800 µL of methanol, exhibiting high absorbance at 517 nm. Methanolic solutions of CMFE, CMFE@CuO NPs, and gallic acid (standard) were prepared at initial concentrations of 1 mg mL^−1^ and serially diluted to obtain concentrations ranging from 12.5 to 400 µg mL^−1^. For the assay, 800 µL of each test solution was mixed with 1.2 mL of the DPPH solution and incubated in the dark for 30 minutes. A visible color change from deep violet to pale yellow upon nanoparticle addition indicated effective radical scavenging. Following incubation, absorbance was measured at 517 nm, and the percentage radical scavenging activity and IC_50_ values were calculated using standard equations (eqn (3) and (4)) as follows:3
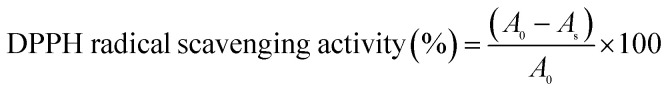
4
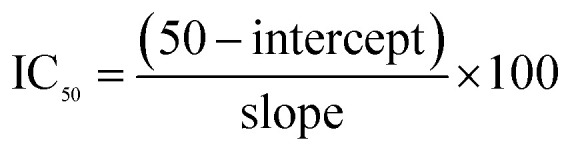
where *A*_0_ and *A*_s_ show the absorbances of the blank and sample solution, respectively.

### 2.11. Statistical analysis

Triplicate experiments were performed to statistically evaluate the results by ANOVA. Accepted results having significance levels of *p* less than 0.05 were reported as the mean ± standard deviation.

## 3. Results and discussion

### 3.1. Phytochemical screening of CMFE

Phytochemical analysis indicated the rich presence of various phenolic compounds, terpenoids, saponins, anthraquinones glycosides and sugars in the *C. macrocarpa* aqueous extract. The results are illustrated in Table S2. The bioactive-rich composition of the fruit extract suggests its potential to reduce metal NPs because bioactives, especially phenolics, play a major role in reduction because of the presence of abundant –OH groups. Hence, this extract was used for the synthesis and capping of CuO NPs.

### 3.2. UV-vis and FTIR analyses

**Fig. 1:**
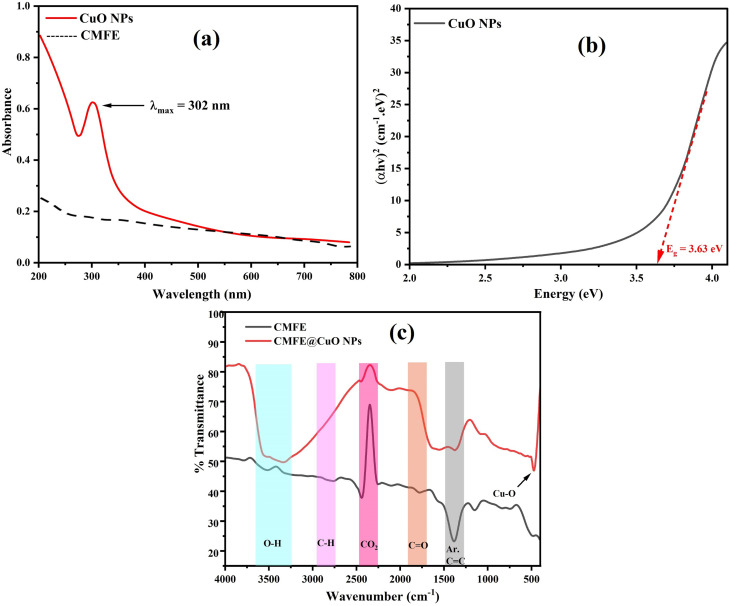
(a) UV-visible absorption spectra of the *C. macrocarpa* fruit extract and synthesized CuO NPs. (b) Tauc plot used to estimate the optical band gap of CuO NPs. (c) FTIR spectra of CMFE and CMFE@CuO NPs.

The UV-vis absorption spectra (Fig. 1a) of the CMFE@CuO NPs sonicated for 15 min exhibited the typical peak of CuO NPs at 302 nm, without a discernible peak for the CMFE. This distinct peak was attributed to the cumulative oscillations of free electrons at the surface of the CuO NPs. In addition, the absorbance data was utilized to construct Tauc plot, which enabled the bandgap energy to be calculated using eqn (5) (ref. 42) as follows:

5 (*αhν*)^1/*n*^ = *B*(*hν* − *E*_g_)^*n*^

where ‘*hν’* is the photon energy, ‘*α*’ is the molar absorption coefficient, and ‘*h*’ represents Planck's constant, and *ν* and *B* correspond to the frequency and a constant of proportionality, respectively. By plotting the graph, the direct energy bandgap (*E*_g_) was found to be 3.63 eV, as shown in Fig. 1b. The observed large *E*_g_ value reflects the catalyst's ability to respond to sunlight for the generation of charge carriers (electron–hole pairs) and was likely due to intragap states in the lattice and quantum confinement effects.^51^

The FTIR analysis confirmed the successful coating of the NPs with various bioactives through the identification of multiple functional groups on the surface of the NPs and in the extract. As evidenced by the FTIR spectra (Fig. 1c) of CMFE and CMFE@CuO NPs, the corresponding functional group peaks were found at 1450 cm^−1^ (C=C stretching) for aromatic compounds, 1690 cm^−1^ (C=O stretching) for carbonyl-containing phytochemicals, 2310 cm^−1^ for CO_2_, 2940 cm^−1^ (C–H stretching) for aliphatic compounds, and 3250–3600 cm^−1^ (O–H stretching) for phenolics and adsorbed moisture.^52^ The existence of such peaks demonstrated that the surface of the NPs had been well coated. Additionally, a detectable peak around 500 cm^−1^, indicative of Cu–O stretching, confirmed metal oxide formation in the crystal lattice.

### 3.3. PXRD, TEM and EDX analyses

**Fig. 2:**
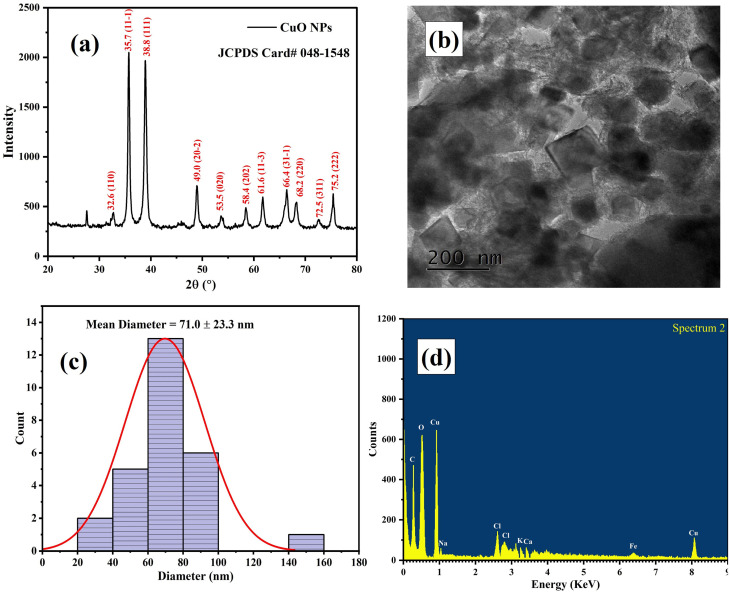
(a) PXRD pattern of the green-synthesized CMFE@CuO NPs. (b) TEM image illustrating the morphology of CMFE@CuO NPs. (c) Particle-size distribution histogram of CMFE@CuO NPs. (d) EDX spectrum of CMFE@CuO NPs.

The PXRD diffractogram of the CuO NPs depicted in Fig. 2a exhibited typical diffraction peaks at 2*θ* values of 32.6°, 35.7°, 38.8°, 49.0°, 53.5°, 58.4°, 61.6°, 66.4°, 68.2°, 72.5°, and 75.2°. These strong Bragg's reflections were indexed to (110), (11-1), (111), (20-2), (020), (202), (11-3), (31-1), (220), (311), and (222) lattice planes, respectively, confirming the monoclinic phase of CuO NPs in accordance with JCPDS card no. 048-1548. Based on diffraction data, the average crystallite size and other strain parameters, as shown in Table 1, were determined by applying the equations given in SI S1.^53^

**Table 1:** Summary of the crystallographic lattice parameters of CMFE@CuO NPs

Sample	Average crystallite size ‘*D*’ (nm)	Dislocation density *δ* × 10^−3^ (nm^−2^)	Micro strain *ε* × 10^−3^	Degree of crystallinity (%)
CMFE@CuO NPs	16.9	3.5	0.6	98.9

Scanning electron microscopy (SEM), transmission electron microscopy (TEM), and energy-dispersive X-ray spectroscopy (EDX) were used to analyze the morphology and elemental composition of the CMFE@CuO NPs, respectively. NPs with size dimensions in the nano range were clearly visible in the scanning electron microscopy image (Fig. S3). A TEM image illustrated in Fig. 2b was recorded to examine the form and particle size of the prepared NPs. Cubic NPs were seen in the TEM image, and the particle-size distribution histogram (Fig. 2c) revealed an average particle size of 71.0 ± 23.3 nm. The variation in particle size calculated using the TEM images and PXRD analysis was observed, indicating the polycrystalline nature of the CMFE@CuO NPs. Based on the PXRD analysis, the calculated crystallite size (16.9 nm) was significantly smaller than the particle dimensions observed in TEM images, suggesting that the cubic structures are composed of multiple crystallites rather than being single-crystal particles. Additionally, the broadened XRD diffraction peaks indicated nanoscale crystalline domains formed by polycrystalline aggregates through oriented growth and agglomeration during synthesis. The EDX spectrum of the CMFE@CuO NPs (Fig. 2d) displayed distinguished peaks of Cu at 0.94 and 8.05 keV, along with a prominent peak of oxygen at 0.52 keV, showing Cu and O as the principal constituents. Some weaker peaks for C, Na, Cl, K, and Fe were also detected, probably stemming from extract-derived metabolites or residual contaminants. Typically, these extra peaks are often seen in greenly synthesized NPs.^21^

### 3.4. DLS and ZP analyses of CMFE@CuO NPs

**Fig. 3:**
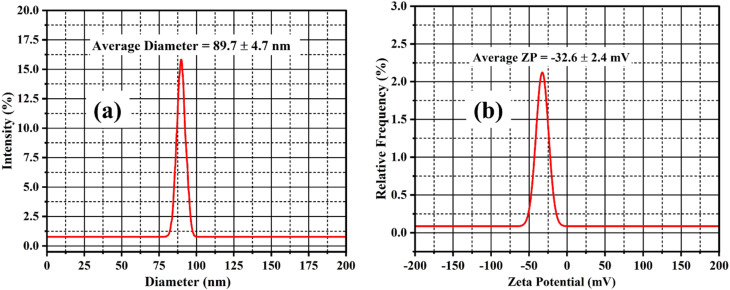
(a) Hydrodynamic radius measurement of CMFE@CuO NPs by DLS analysis. (b) Zeta potential profile of CMFE@CuO NPs.

ZP and DLS analyses were used to evaluate the surface charge and hydrodynamic size of the CMFE@CuO NPs. The DLS results (Fig. 3a) showed a particle-size distribution ranging from 77 to 98 nm, with an average hydrodynamic diameter of 89.7 ± 4.7 nm. The particle size obtained from TEM analysis was comparatively smaller, which is expected because of the absence of solvent effects. This difference arises because DLS measurements account for the hydration layer formed around NPs when dispersed in aqueous media, resulting in a larger apparent particle size.

The magnitude of the negative average ZP value of −32.6 ± 2.4 mV (Fig. 3b) depicts the good stability of the CuO NPs on capping with the phytochemicals of the CMFE involved in the green synthesis of NPs. According to the ZP results, the suspension exhibited remarkable electrostatic stability, allowing NPs to remain dispersed for a long time with minimal agglomeration.

### 3.5. Thermogravimetric analysis of CMFE@CuO NPs

**Fig. 4:**
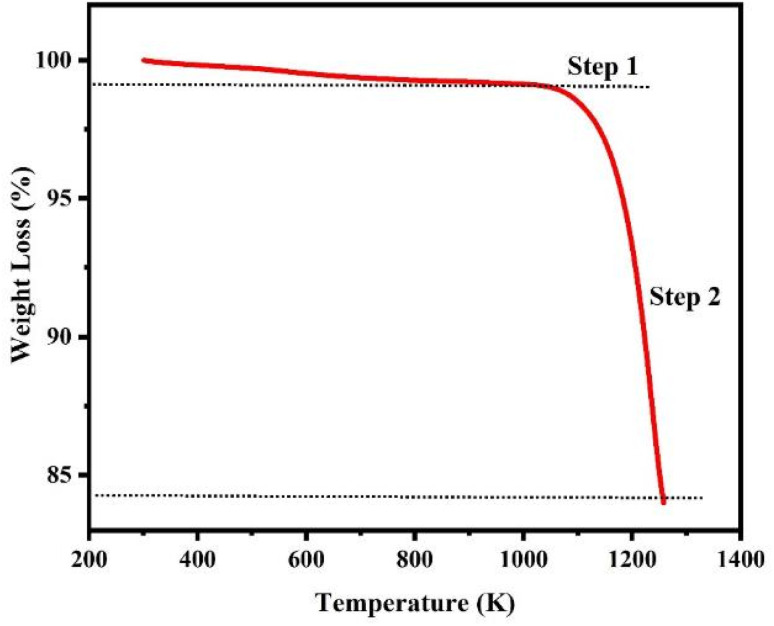
Thermogravimetric plot of CMFE@CuO NPs.

TGA analysis using a thermal analyzer (Discovery 650 SDT, TA Instruments, USA) was conducted to assess the thermal stability of the prepared CuO NPs within a temperature range of 25–1000 °C, as shown in Fig. 4. The findings demonstrated strong concordance with the configuration and decomposition procedure. Thermal degradation occurred in two stages: small weight loss (∼1.2%) in the range of 300–1084 K due to water evaporation and moisture loss and 14.8% weight loss in the range of 1084–1250 K due to the decomposition of organic moieties. Overall, the sample showed high stability (only 16% weight loss) even at high temperatures.

### 3.6. Photodegradation of MB

Owing to their optimal band gap of 3.63 eV, the prepared CMFE@CuO NPs were investigated for their photocatalytic potential against the sunlight-assisted degradation of MB dye. The large *E*_g_ value allows the catalyst's valence electrons to absorb sunlight, facilitating their transfer from the VB to the CB and leading to the formation of excitons (electron–hole pairs). The UV-vis spectra recorded at regular time intervals indicated a progressive reduction in the dye concentration, as evidenced by a steady decrease in the MB solution's absorption intensity at *λ*_max_ (668 nm) up to 120 minutes, as illustrated in Fig. S4.

### 3.7. RSM/BBD for the optimization of MB degradation

The RSM/BBD model was used to observe how four distinct factors and their correlation affected the photodecomposition of MB. Table 2 displays the findings of the BBD model from 29 experimental trials, including the expected and actual values. These statistics validate the quadratic model's appropriateness for MB degradation. The elevated adjusted *R*^2^ (0.9856) suggests a robust model fit. The minimal lack-of-fit *p*-value (0.8471), along with a significant sequential *p*-value (<0.0001) and the low standard deviation of 2.72, signifies model adequacy and good prediction accuracy. Table S3 describes the model's fit as proposed by the software, and Fig. S5 indicates a strong correlation between the experimental and anticipated results.

**Table 2:** Box–Behnken design for the optimization of the four parameters influencing the degradation of MB

Run	*A*: pH	*B*: temperature (K)	*C*: concentration of dye (ppm)	*D*: catalyst dosage (mg)	MB dye degradation (%)	
					Actual value	Predicted value
1	3	328	30	30	57	60.4
2	13	298	20	30	36.5	37.0
3	8	328	20	30	92.5	90.0
4	13	328	30	30	29	29.3
5	8	298	20	50	88	88.3
6	13	328	20	50	38	37.6
7	8	298	20	10	80.5	79.7
8	8	358	10	30	94	94.2
9	8	328	20	30	92	90
10	13	328	20	10	31	31.7
11	8	328	20	30	90.5	90.0
12	8	328	10	10	84	83.8
13	8	328	30	10	74	74.8
14	3	328	20	50	68	65.7
15	8	358	20	10	75	75.4
16	3	358	20	30	58	58.1
17	3	298	20	30	56	55.3
18	8	328	20	30	84	90.0
19	13	358	20	30	37	38.2
20	3	328	20	10	43	41.8
21	8	298	10	30	91	93.2
22	13	328	10	30	52	49.3
23	8	328	10	50	98	97.7
24	3	328	10	30	56	56.4
25	8	358	20	50	95	96.6
26	8	298	30	30	86	84.2
27	8	358	30	30	91	87.2
28	8	328	20	30	91	90.0
29	8	328	30	50	90	90.7

Moreover, ANOVA was conducted to validate the importance and relevance of the BBD-proposed model, and the outcomes are depicted in Table 3. The *F*-value of 138.24 suggests the model's significance with a 0.01% chance that the noise may have caused this large value.

**Table 3:** ANOVA results of the quadratic polynomial model for MB degradation

Source	Sum of squares	df	Mean square	*F*-value	*p*-value	Remarks
Model	14 310.44	14	1022.17	138.24	<0.0001	Significant
*A*-pH	1092.52	1	1092.52	147.75	<0.0001	
*B*-temperature	12.00	1	12.00	1.62	0.2234	
*C*-concentration of dye	192.00	1	192.00	25.97	0.0002	
*D*-catalyst dosage	667.52	1	667.52	90.27	<0.0001	
*AB*	0.5625	1	0.5625	0.0761	0.7867	
*AC*	144.00	1	144.00	19.47	0.0006	
*AD*	81.00	1	81.00	10.95	0.0052	
*BC*	1.0000	1	1.0000	0.1352	0.7186	
*BD*	39.06	1	39.06	5.28	0.0375	
*CD*	1.0000	1	1.0000	0.1352	0.7186	
*A* ^2^	11 340.23	1	11 340.23	1533.64	<0.0001	
*B* ^2^	6.49	1	6.49	0.8772	0.3648	
*C* ^2^	3.65	1	3.65	0.4934	0.4939	
*D* ^2^	100.57	1	100.57	13.60	0.0024	
Residual	103.52	14	7.39	—	—	Not significant
Lack of fit	56.02	10	5.60	0.4718	0.8471	
Pure error	47.50	4	11.88	—	—	
Total	14 413.97	28	—	—	—	

Model significance was confirmed by *P*-values below 0.0500, identifying factors *A*, *C*, *D*, their interaction terms (*AC*, *AD*, *BD*), and quadratic terms (*A*^2^ and *D*^2^) as statistically significant contributors to MB degradation. Insignificant terms with *P*-values greater than 0.1 were excluded from the model to improve predictive accuracy. The lack-of-fit *F*-value (0.47) was insignificant relative to the pure error, indicating good model adequacy. A regression equation expressed in coded variables (eqn (6)) was employed to assess the individual and interactive effects of process parameters on degradation efficiency as follows.

6 MB degradation(%) = 90 − 9.5*A* + *B* − 4*C* + 7.46*D* − 0.3750*AB* − 6*AC* − 4.5*AD* + 0.5*BC* + 3.13*BD* + 0.5*CD* − 41.81*A*^2^ − *B*^2^ + 0.75*C*^2^ − 3.94*D*^2^

**Fig. 5:**
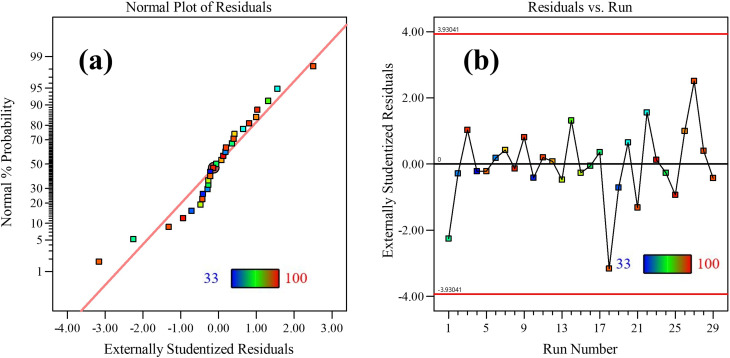
(a) Normal plot of residuals. (b) Residual *vs.* run distribution plot.

The intercept value of 90 represents the baseline response, while the linear coefficients correspond to the direct effects of the independent variables. Quadratic coefficients account for curvature in the response surface, and interaction coefficients describe the combined influence of paired variables. Model validity was further confirmed through residual diagnostics, including normal probability and residual-versus-run plots (Fig. 5a and b). The linear distribution of residuals and their random dispersion around the central axis demonstrate the reliability and robustness of the developed model.

**Fig. 6:**
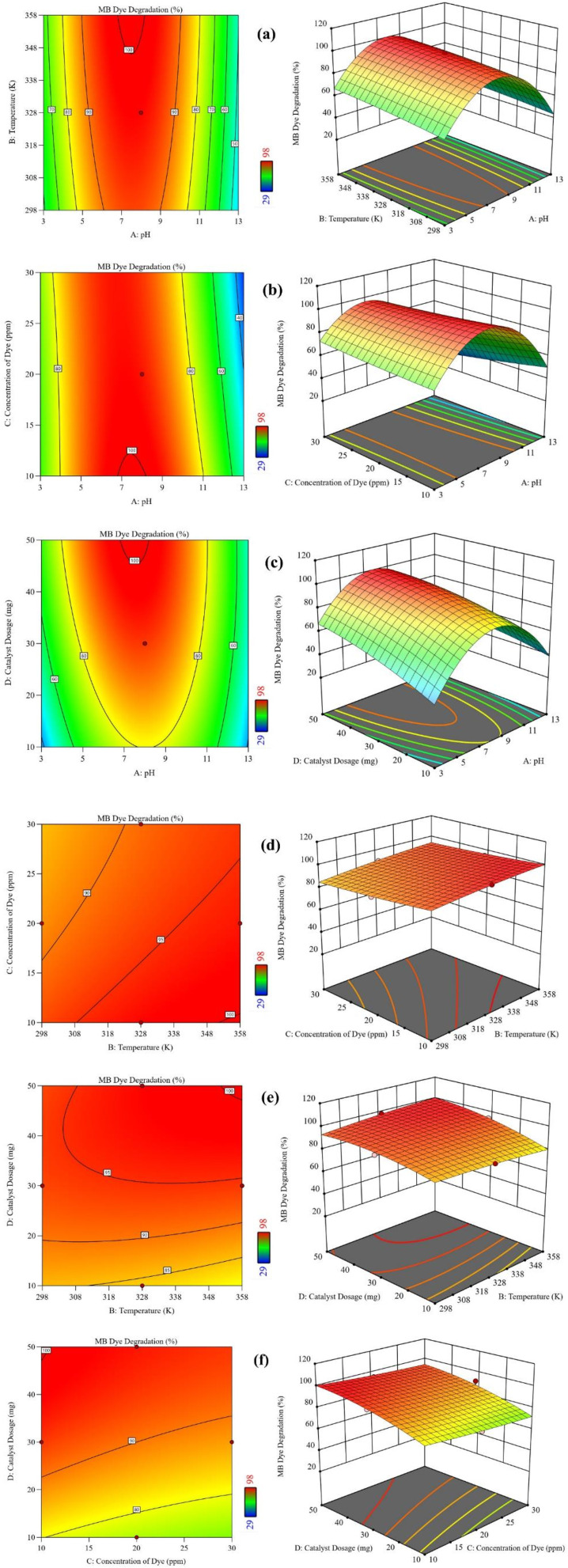
Two-dimensional contour and three-dimensional response plots of the effect parameters: (a) pH of the medium and temperature (K), (b) pH of the medium and initial dye concentration (ppm), (c) pH of the medium and CMFE@CuO NP dosage (mg), (d) temperature (K) and initial dye concentration (ppm), (e) temperature (K) and CMFE@CuO NP dosage (mg), and (f) initial dye concentration (ppm) and CMFE@CuO NP dosage (mg).

2D contour plots and 3D response surface graphs analyze how two factors simultaneously affect the dye degradation efficiency across the given ranges by keeping other factors constant. The 2D contour plots and 3D response surface graphs of various factors are shown in Fig. 6.

Fig. 6a shows that at a pH of 8, an increase in the temperature enhanced the degradation efficiency, and the maximum degradation (98%) was achieved at 328 K, a catalyst dosage of 50 mg, and a dye concentration of 10 ppm. It was also observed that the degradation efficiency could reach 100% at 348 K. Furthermore, the *F*-values of the independent factors indicate that pH had the most tremendous impact on degradation. Since MB is a redox indicator, a pH change significantly impacts the nanomaterial's surface charge, dye's ionic nature, adsorption potential, and band potentials. Its color and *λ*_max_ (668 nm) are not affected by changes in the solution pH. The resonance structures of MB are shown in Fig. S6. However, a low pH can lead to particle agglomeration due to neutralization of surface charge, which, in turn, reduces surface area, diminishes dye adsorption, and limits the overall photocatalytic potential. It was observed that at pH > 9, the CMFE@CuO NPs began to ionize *via* hydrolysis, leading to a significant decrease in the NP concentration and a subsequent decline in the photocatalytic efficiency. However, the degradation efficacy significantly rose at a pH of around 7 because of optimal dye adsorption, but it dropped again at a lower pH as H^+^ ions scavenged the ^−^OH and ˙OH radicals. Thus, effective control of pH is vital for the efficient degradation of organic pollutants.

In addition to pH, catalyst dosage considerably affects the degradation efficiency, as evidenced by the *F*-value. It was shown that increasing the catalyst dose considerably enhanced the degradation efficiency by supplying more reactive sites. Yet, large dosages can limit efficiency since particles agglomerate and cause turbidity in suspension, which, in turn, blocks efficient light penetration. Thus, the greatest photocatalytic efficacy was attained at a pH of 8 with a catalyst dosage of ∼50 mg, as shown in Fig. 6c.

Alongside pH and catalyst dosage, the initial dye concentration was found to be a critical factor impacting degradation efficiency. As demonstrated in Fig. 6b and f, maximal degradation took place at the lowest dye concentration at a pH of 8 with a catalyst dosage of 50 mg. Increasing dye concentration enhanced competition for active sites on the catalyst surface, limiting degradation efficiency over the 120 minutes reaction period due to the limited number of reactive sites. At extremely high dye concentrations, the degradation efficiency dropped further due to lower light penetration, hence inhibiting photocatalytic activity.

Temperature showed a relatively small influence on dye degradation, as illustrated in Fig. 6(d and e). ANOVA indicated that an increasing temperature slightly enhanced the degradation rates by increasing the kinetic energy of the dye molecules, although its overall contribution to efficiency remained limited.

The cumulative effect of variables on the MB degradation (%) was better elaborated by the 3D response plots. The interactive effect of pH and temperature (Fig. 6a) demonstrated that the MB degradation (%) can be enhanced with an increase in temperature and pH, reaching a maximum value (99.9%) at a pH of 8 and 358 K. At the highest temperature, an increase in the pH above 8 decreased the degradation efficiency because of the ionization of the CMFE@CuO NPs and reduction of ROS in the system.

The interactive effect of the initial dye concentration and pH (Fig. 6b) showed an increase in the MB degradation (%) with a decrease in the dye concentration up to 10 ppm and an increase in the pH up to 8. Maximum degradation (98%) was observed at a pH of 8 and a dye concentration of 10 ppm at 328 K. Similarly, the interactive plots of pH and catalyst dose (Fig. 6c), temperature and initial dye concentration (Fig. 6d), catalyst dose and temperature (Fig. 6e), and catalyst dose and initial dye concentration (Fig. 6f) showed that the MB degradation (%) increased with an increase in catalyst dosage (maximum at 50 mg), temperature (up to 358 K), and pH (up to 8), while it decreased at a high dye concentration (above 10 ppm).

**Fig. 7:**
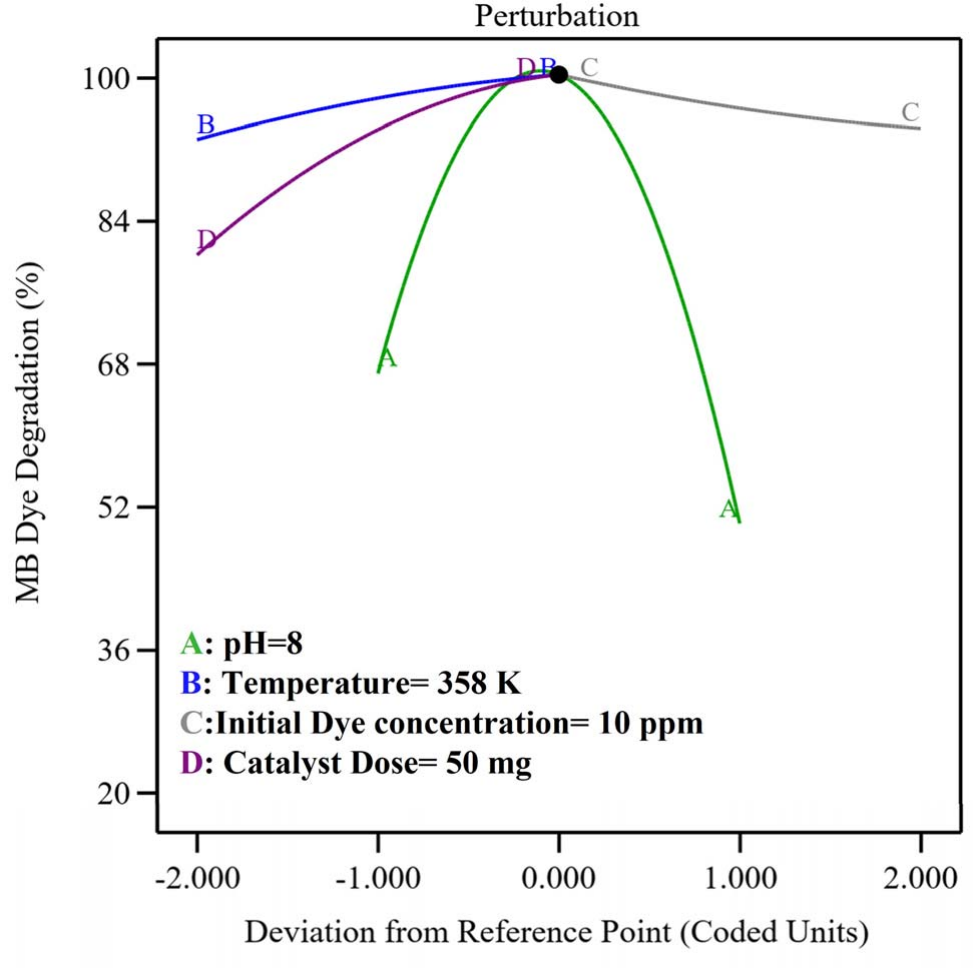
Perturbation plot of the interfering reaction parameters.

Fig. 7 illustrates the collective outcome of the reaction conditions on MB degradation. The perturbation graph demonstrates that degradation (%) decreased when pH deviates from the ideal value of 8, possibly caused by changes in the NP surface charge or ionization. A higher catalyst dose, accompanied by a high temperature, enhanced the degradation efficiency (%) by providing more active sites and kinetic energy to molecules to overcome the energy barrier, respectively. However, dye degradation (%) increased with a decrease in the dye concentration (ppm) due to greater light penetration into the cell.

The optimization of reaction parameters indicated that the maximum degradation efficiency (99.9%) was achieved under relatively milder conditions (50 mg of catalyst, 10 ppm dye, pH of 8, and temperature of 358 K), suitable for commercial applications of synthesized CMFE@CuO NPs. A pH of 8 indicated that the process operates effectively under near-neutral to slightly alkaline conditions, minimizing the need for extensive pH adjustment in real wastewater systems. The optimized dye concentration of 10 ppm, characteristic of diluted industrial effluents, indicated the process's potential for wastewater treatment. A moderate catalyst dose of 50 mg emphasized cost-effectiveness by minimizing material use and secondary waste. The optimal operational temperature of 358 K implied that the process can be conducted at manageable temperatures, achievable in practical situations without significant energy requirements. These optimized parameters collectively affirm the feasibility, economic viability, and environmental compatibility of the proposed catalytic system for application in real-world wastewater management.

**Fig. 8:**
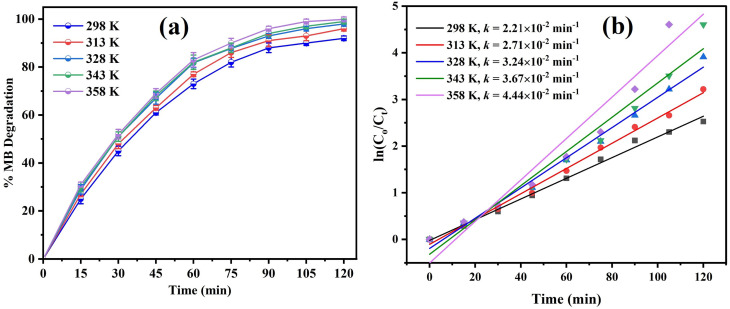
(a) Time-dependent degradation of MB. (b) Effect of temperature on the rate constant values.

Degradation kinetics were evaluated using eqn (1) at 15 minutes intervals for a total duration of 120 minutes, utilizing the absorbance spectra of MB, as illustrated in Fig. 8a. The percentage degradation of MB increased slightly with an increase in the reaction medium temperature, reaching a maximum of 99.9% at 358 K. The kinetics models (zero-order, pseudo-first-order, and pseudo-second-order) were applied to examine the degradation kinetics using eqn (7)–(9) as follows:

7 *C*_*t*_ = *C*_0_ − *k*_0_^*t*^

8
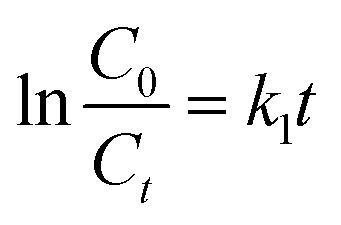
9
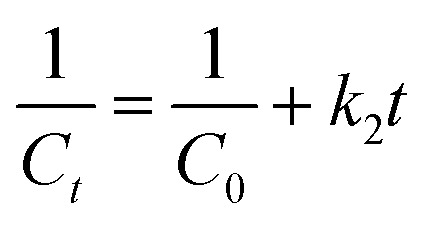


*C*_0_ and *C*_t_ are the initial dye concentration and dye concentration at any time *t*, respectively, and *k*_0_, *k*_1,_ and *k*_2_ are dye degradation rate constants for the zero, first, and second-order kinetic models, respectively. The dye degradation data was fitted to the zero-, first-, and second-order reaction kinetic models. The fitting of data to the zero-, first- and second-order kinetic models is presented in Fig. S7(a–c). The values of regression coefficients (*R*^2^) for the zero-, first- and second-order reaction kinetic models were found to be 0.871, 0.990, and 0.837, respectively. The results indicate that the dye degradation process obeyed the first-order kinetics. Therefore, the values of rate constants at different temperatures were determined using eqn (8). The slopes of the degradation plots (Fig. 8b) give the degradation rate constant (*k*) values of 2.21 × 10^−2^, 2.71 × 10^−2^, 3.24 × 10^−2^, 3.67 × 10^−2^ and 4.44 × 10^−2^ min^−1^ at 298, 313, 328, 343, and 358 K, respectively. Compared with previously reported catalysts, as listed in Table S2, the current CuO NPs demonstrated superior efficacy in the degradation of MB.

### 3.8. Effect of radical scavengers and proposed mechanism

ROS play a central role in the rapid degradation of organic pollutants.^54^ To elucidate the photocatalytic degradation mechanism and identify the dominant reactive species, batch-wise radical scavenging experiments were performed. *P*-benzoquinone (*p*-BQ), isopropanol (IPA), disodium ethylenediaminetetraacetate (Na_2_EDTA), and l-ascorbic acid (l-AA) were employed as selective scavengers for O_2_˙^−^, ˙OH, h^+^, and H_2_O_2_, respectively.^55^ Each experiment was performed under optimal conditions (50 mg/20 mL catalyst dose, pH of 8, 10 ppm dye solution) with the addition of 10 mL of a 0.2 mM scavenger solution. The degradation efficiency (%) was calculated using the abovementioned procedure.

**Fig. 9:**
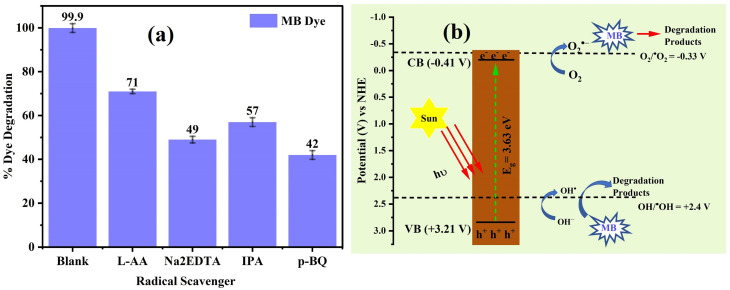
(a) Degradation of MB in the presence of radical scavengers. (b) Band edge potentials of CMFE@CuO NPs for the generation of ROS in the presence of sunlight to degrade the MB dye.

Radical-scavenging assays showed that the decomposition efficiency diminished in the presence of all scavengers, as shown in Fig. 9a. This result showed that all ROS were produced in the solution as a result of sunlight exposure. The degradation phenomena were most significantly prevented in the presence of *p*-BQ, followed by Na_2_EDTA and IPA. This implies that h^+^ and OH˙ played secondary roles in the degradation process, but O_2_^−^˙ was an active participant.

The Butler–Ginley equations (S3 of SI) were used to calculate the edge potentials of the VB and CB in order to attain a thorough grasp of the electron transport and degradation mechanisms.^56^

The values of *E*_CB_ and *E*_VB_ were determined to be −0.41 and +3.21 V, respectively, as illustrated in Fig. 9b. The viable reduction and oxidation potentials enable the easy formation of reactive oxygen species (ROS), including O_2_^−^˙, h^+^, and OH˙, at the surface of the catalyst in solution, as depicted in Fig. 9b.

The small potential difference for electron transfer to molecular oxygen facilitated the production of oxide ion radicals, highlighting their role as principal species involved in degradation reactions.

The catalytic decomposition pathway of MB dye was postulated by studying the influence of radical scavengers on the process, as presented in S3 of the SI. It was hypothesized that under sunlight irradiation, the catalyst's valence electrons would be stimulated to the conduction band, resulting in the formation of e^−^ and h^+^ in the CB and VB. Oxide ion radicals were formed by the reaction of O_2_ with e^−^ in the CB, and OH˙ radicals were formed by the reaction of h^+^ with water. Dye degradation involves the production of a variety of secondary radicals in the mixture as a result of secondary reactions.

### 3.9. TOC analysis results

**Fig. 10:**
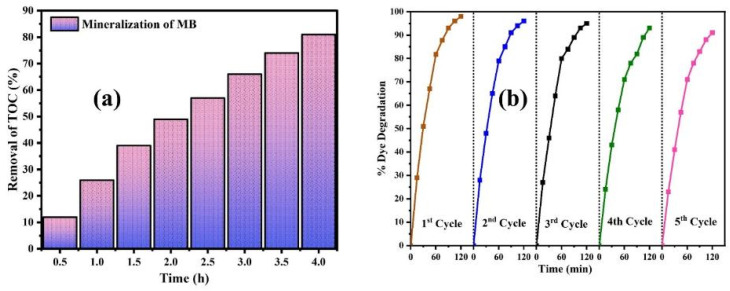
(a) Percentage mineralization of MB at different time intervals during the photocatalytic degradation. (b) Reusability performance of CMFE@CuO NPs over successive degradation cycles.

The estimated TOC values showed that dye mineralization was a sluggish phenomenon compared to color disappearance, with 81% mineralization of the MB dye under sunlight illumination for 4 h, as shown in Fig. 10a. This slow rate is attributed to the gradual breakdown of stable components and by-products formed during photodegradation.

### 3.10. Reusability analysis of the photocatalyst

**Fig. 11:**
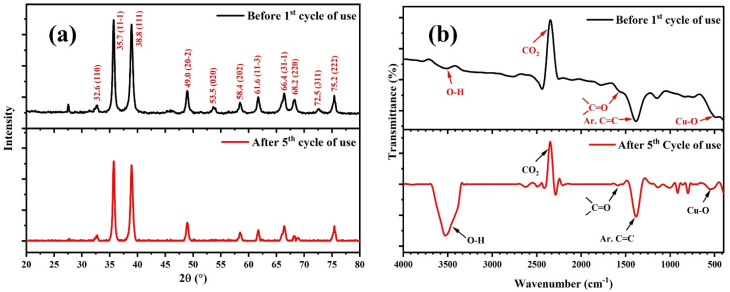
(a) Comparative PXRD spectra of the freshly prepared CMFE@CuO NPs and after five uses. (b) Comparative FTIR spectra of the freshly prepared CMFE@CuO NPs and after five uses.

The reusability of the photocatalyst was tested for five consecutive cycles of MB dye degradation using a previously reported method.^21,57^ The catalyst, namely the CMFE@CuO NPs, was extracted by spinning the solution at 3000 rpm for 30 minutes. The solid catalyst was dried for four hours at 80 °C and then rinsed three times with distilled water to make it suitable for reuse. Fig. 11b shows the catalyst's reusability with minimal activity loss. After several repeated cycles, the catalyst retained most of its activity with only a 7% drop, showing its durability and effectiveness for repeated usage. The mechanical stability of the catalyst was also assessed by recording the comparative PXRD and FTIR spectra of the catalyst after five uses, as depicted in Fig. 11a and b. After five consecutive uses, the PXRD spectrum of regenerated CMFE@CuO NPs (Fig. 11a) retained all its distinctive peaks, suggesting that the crystalline structure of the catalyst remained intact. However, a slight increase in crystallite size was observed from 16.9 to 18.9 nm after regeneration. The values of the crystallite parameters depicted in Table 4 indicate the catalyst's high mechanical stability for practical applications. Similarly, the comparison with the FTIR spectra (Fig. 11b) showed the chemical stability of the catalyst, as all the peaks of the functional groups were retained in the regenerated catalyst. The only hydroxyl peak (–OH) around 3300–3600 cm^−1^ was observed to become more prominent in the regenerated catalyst, which can be due to the adsorbed moisture on the surface of the catalyst. Hence, the catalyst presented appears to be suitable for multiple uses in wastewater treatment after proper activation.

**Table 4:** Crystal parameters of CMFE@CuO NPs after the 5th cycle of use

Sample	Average crystallite size ‘*D*’ (nm)	Dislocation density *δ* × 10^−3^ (nm^−2^)	Micro strain *ε* × 10^−3^	Degree of crystallinity (%)
CMFE@CuO NPs	18.9	2.7	0.59	95.2

The leaching of heavy metal ions, like Cu^2+^, into freshwater reservoirs may affect the quality of life of aquatic animals. An elevated level of Cu^2+^ in freshwater may induce oxidative stress in aquatic animals. Therefore, the leaching of Cu^2+^ ions from the surface of the CMFE@CuO NPs catalyst was quantified by atomic absorption spectroscopy (AAS).^58^ For this purpose, after each degradation cycle, the reaction mixture was centrifuged, and the filtrate was analyzed using a graphite furnace atomic absorption spectrometer (AA-6300, Shimadzu Japan). The results indicated that the concentration of leached Cu^2+^ ions was just 8 ppm after the 5th cycle of use, demonstrating minimal dissolution of the catalyst (only 0.32%).

This low level of copper leaching (below 1%) highlighted the highly stable and reusable nature of greenly synthesized CMFE@CuO NPs, supporting the reusability analysis results and post-usage PXRD and FTIR analysis of the catalyst. The low leaching level of the catalyst also emphasized that the MB dye degradation reaction was predominantly driven by heterogeneous catalysis rather than homogeneous contributions from dissolved Cu^2+^ ions. All these analysis results highlighted the consistent degradation efficiency of the greenly synthesized CMFE@CuO NPs over multiple cycles and their suitability for repeated wastewater treatment applications.

### 3.11. Environmental implications and safe disposal strategies of CMFE@CuO NPs

The presence of excessive leached Cu^2+^ ions in the treated water may affect aquatic animals if the effluent is released directly into freshwater bodies. Although the reported catalyst has shown minimal Cu^2+^ leaching (0.32%) after several cycles and can be used to treat wastewater, the recovery of leached Cu^2+^ ions is still essential for sustainable applications. To minimize the environmental implications and achieve the safe disposal of the catalyst, the following steps must be taken.

(1). The CMFE@CuO NPs must be efficiently recovered after each cycle by centrifugation to minimize environmental discharge.

(2). The treated effluent should be monitored to ensure that residual copper levels comply with environmental regulations before disposal. Excess Cu^2+^ should be removed by additional operations, such as adsorption, ion exchange, or precipitation.^59^

(3). If the catalyst becomes inactivated after several uses, it should be collected and either regenerated for reuse or disposed of according to hazardous waste management guidelines.

### 3.12. Antibacterial and antioxidant activities of CMFE@CuO NPs

**Fig. 12:**
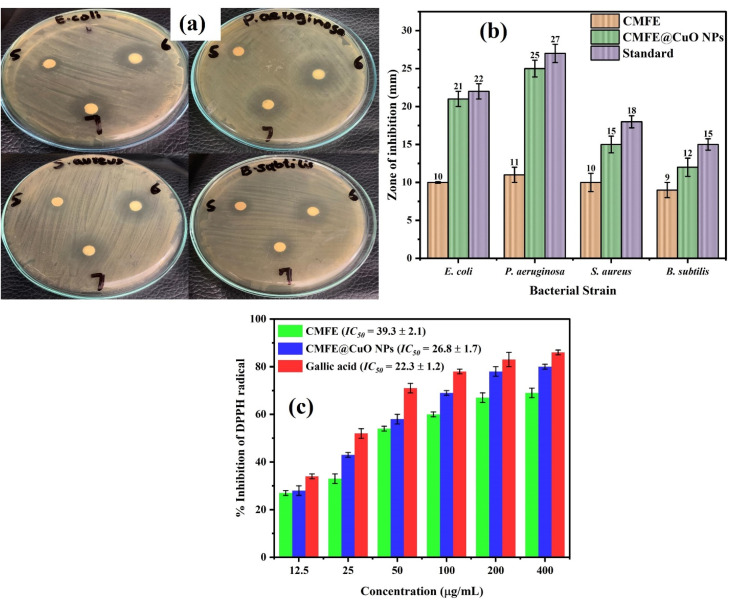
(a) Images of the disc diffusion assay showing the zone of inhibition of CMFE (5), positive control (6) and CMFE@CuO NPs (7) against bacterial strains. (b) Zones of inhibition exhibited by CMFE, CMFE@CuO NPs, and erythromycin (standard) against different bacterial strains. (c) Antioxidant activity of CMFE, CMFE@CuO NPs, and gallic acid (standard).

The disc diffusion assay assesses the antibacterial potential of CMFE and CMFE@CuO NPs against *S. aureus*, *B. subtilis*, *E. coli*, and *P. aeruginosa* bacterial strains. CMFE exhibits strong antibacterial potential.^20^ Antimicrobial testing was conducted on the samples to ascertain the synergistic effect of NPs mixed with bioactive capping agents. Encapsulating NPs with phytochemical compounds exerted a synergistic effect, markedly enhancing their antibacterial efficacy. Fig. 12a illustrates the antibacterial efficacy of CMFE in comparison to the CMFE@CuO NPs.

The zone of inhibition results demonstrated that the CMFE@CuO NPs exhibited greater potency in inhibiting bacterial growth than CMFE (Fig. 12b). The CMFE@CuO NPs showed increased sensitivity to Gram-negative bacterial strains, as evidenced by large inhibition zones against *E. coli* (21 ± 1.0 mm) and *P. aeruginosa* (25 ± 1.1 mm), as compared to Gram-positive bacteria, *i.e.*, *S. aureus* (15 ± 1.1 mm) and *B. subtilis* (12 ± 1.2 mm). The activity of CMFE@CuO NPs against Gram-negative bacteria was found to be in close agreement with the standard drug, erythromycin. The MIC values of the CMFE, CMFE@CuO NPs, and standard are depicted in Table 5.

**Table 5:** MIC values of CMFE and CMFE@CuO NPs against bacterial strains

Sample	MIC value (µg mL^−1^)			
	*E. coli*	*P. aeruginosa*	*S. aureus*	*B. subtilis*
CMFE	315 ± 4.5	289 ± 3.2	322 ± 2.7	342 ± 5.2
CMFE@CuO NPs	118 ± 2.3	103 ± 4.1	182 ± 3.5	217 ± 3.3

The precise mechanism of action remained unidentified; however, numerous studies indicate that NPs exert their effects by infiltrating bacterial cells, generating ROS both intracellularly and extracellularly, producing metal ions within the cell, inactivating enzyme active sites, and irreversibly binding to genetic material. All these interactions culminate in cell death, either by the rupture of the cell wall and subsequent plasma discharge or by impairing the proper functioning of the cells. The exceptional efficacy of the CMFE@CuO NPs against Gram-negative bacteria was attributed to differences in the bacterial cell membranes.^21,60,61^ Gram-negative bacteria have thin cell walls, which make it possible for CMFE@CuO NPs to enter and disrupt cellular functions, thereby increasing antibacterial activity.

Gallic acid was used as a standard because of its well-known capacity to neutralize DPPH radicals by hydrogen donation. The DPPH radical-scavenging test was used to assess the antioxidant capability of the synthesized samples. The presence of hydroxyl groups, which are well known for their potent antioxidant properties, is responsible for this action. The evaluation of the antioxidant ability of CMFE and CMFE@CuO NPs was further supported by the discovery of –OH functional groups in the FTIR spectra of the samples.

As shown in Fig. 12c, the DPPH assay findings revealed a concentration-dependent increase in antioxidant activity for CMFE, CMFE@CuO NPs, and gallic acid. The CMFE@CuO NPs outperformed the standard in terms of antioxidant efficacy at higher doses (400 µg mL^−1^). The quantity of hydroxyl-rich phytoconstituents covering the surface of the NPs is responsible for this increased activity. This pattern was further supported by the IC_50_ values, which showed that CMFE@CuO NPs had a higher radical-scavenging efficiency (IC_50_ = 26.8 ± 1.7 µg mL^−1^) than CMFE (IC_50_ = 39.3 ± 2.1 µg mL^−1^). Furthermore, the IC_50_ value of CMFE@CuO NPs was similar to that of gallic acid (22.3 ± 1.2 µg mL^−1^), highlighting their strong antioxidant potential.

For further validation of the importance of the synthesized CMFE@CuO NPs, a comparison of the current work has been depicted in a table (Table 6). The comparison table clearly demonstrates the highly bioactive nature of the synthesized material to inhibit bacterial growth and act as an antioxidant for wastewater treatment.

**Table 6:** Comparison table of the antioxidant potential of greenly synthesized CuO NPs

Material	Extract	Antioxidant activity by DPPH assay (IC_50_ (µg mL^−1^))	Antibacterial activity (strain (ZOI))	Reference
CuO NPs	*Solanum nigrum* leaf	189.12	*E. coli* (12 ± 0.1 nm), *B. subtilis* (11 ± 0.3 nm), *S. saprophyticus* (10 ± 0.2 nm) and *P. aeruginosa* (8 ± 0.5 nm)	62
CuO NPs	*Tribulus terrestris L*	51.53	*S. aureus* (17 mm) and *E. coli* (18 mm)	63
CuO NPs	*Ligustrum lucidum*	63.35	—	64
CuO NPs	*Vernonia amygdalina*	—	*E. coli* (12 mm), *P. aeruginosa* (12 mm), and *E. aerogenes* (15 mm)	65
CuO NPs	*Suaeda maritima* (L.) Dumort	28.05	*B. subtilis* (17.1 mm), *S. aureus* (16.5 mm), *E. coli* (14.3 mm), and *P. aeruginosa* (15.8 mm)	66
CuO NPs	Fruit waste	—	*E. coli* (29.0 ± 2.3 mm) and *S. aureus* (26.0 ± 1.1 mm)	67
CMFE@CuO NPs	*C. macrocarpa* fruit	26.8 ± 1.7	*E. coli* (21 ± 1.0 mm) and *P. aeruginosa* (25 ± 1.1 mm), *S. aureus* (15 ± 1.1 mm) and *B. subtilis* (12 ± 1.2 mm)	Current work

Although the greenly synthesized CMFE@CuO NPs exhibit appreciable antimicrobial and antioxidant potential, the *in vivo* use of this material is not recommended because of cytotoxicity concerns. However, the results have indicated that this material can be effectively used to purify wastewater from wastewater-borne microbes and can be used in creams and ointments for external use only.

## 4. Conclusion

The biogenic synthesis of CuO NPs based on natal plum aqueous extract is presented in this work. With an average particle size of 71 nm and a crystallite size of 16.9 nm, the CMFE@CuO NPs demonstrated a broad bandgap of 3.63 eV, making them appropriate for the production of charge carriers under sunlight on the surface of the catalyst. The CMFE@CuO NPs demonstrated an outstanding photodegradation of MB (99.9%) at a pH of 8, 50 mg of catalyst, and 10 ppm dye concentration at 358 K, following reaction parameter optimization using the RSM/BBD model. O_2_^−˙^ showed the primary contribution towards the degradation process, followed by h^+^ and OH^˙^ radicals, according to radical-scavenging assays. These results were found to be concordant with the band edge potentials of CMFE@CuO NPs measured to evaluate the generation of major ROS produced. Regeneration studies of the catalyst showed a mere 7% decline in the catalyst's efficiency even after being used five times. The CMFE@CuO NPs were also found to be effective antibacterial agents in inhibiting the growth of Gram-negative bacteria (*E. coli* (21 ± 1 mm) and *P. aeruginosa* (25 ± 1.1 mm)) as compared to Gram-positive bacteria (*S. aureus* (15 ± 1.1 mm) and *B. subtilis* (12 ± 1.2 mm)). In addition, the CMFE@CuO NPs showed high antioxidant potential (IC_50_ = 26.8 ± 1.7 µg mL^−1^), rivaling that of standard gallic acid (IC_50_ = 22.3 ± 1.2 µg mL^−1^), thereby emphasizing their remarkable potential for industrial wastewater remediation.

## Author contributions

A. B. Siddique: methodology, investigation, writing – original draft, and writing – review and editing. Y. Zaman and A. Abbas: data curation and writing – review and editing. M. F.U. Rehman and M. Sher: writing – review and editing. U. Nishan and Ibrahim A. Shaaban: resources and writing – review and editing.

## Conflicts of interest

The authors have no known financial or non-financial interests to disclose.

## Supplementary Material

NA-OLF-D5NA01159K-s001

## Data Availability

All evaluated data is available in the manuscript. Additional information/data can be provided upon reasonable request. Supplementary information (SI) is available. See DOI: https://doi.org/10.1039/d5na01159k.
