# Supplementary material for: Green synthesis of cubic CuO nanoparticles for biomedical applications and the photodegradation of methylene blue: RSM-BBD optimization of the reaction parameters and stability studies
Source: Nanoscale Adv. 2026 Apr 28;8(11):3396–413. doi: 10.1039/d5na01159k (PMC13169288; doi:10.1039/d5na01159k)
Supplement: NA-008-D5NA01159K-s001 [file NA-008-D5NA01159K-s001.pdf]

Supplementary Information (SI) for Nanoscale Advances.
This journal is © The Royal Society of Chemistry 2026

Green synthesis of cubic CuO nanoparticles for biomedical applications and photodegradation of methylene blue: RSM-BBD optimization of reaction parameters, and stability studies

Abu Bakar Siddique,[a,*] Azhar Abbas,[a,b] Muhammad Sher,[a] Yasir Zaman,[c] Muhammad Fayyaz ur Rehman,[a] Umar Nishan,[d] Ibrahim A. Shaaban [e]

[a] *Institute of Chemistry, University of Sargodha, Sargodha 40100, Pakistan*

[b] *Department of Chemistry, Government Ambala Muslim College, Sargodha 40100, Pakistan*

[c] *Department of Physics, University of Sargodha, Sargodha 40100, Pakistan*

[d] *Department of Chemistry, Kohat University of Science and Technology, Kohat 26000 KP, Pakistan*

[e] *Department of Chemistry, Faculty of Science, Research Center for Advanced Materials Science (RCAMS), King Khalid University, P.O. Box 960, Abha, 61421, Saudi Arab*

* *Corresponding authors*: *abubakar.siddique@uos.edu.pk*

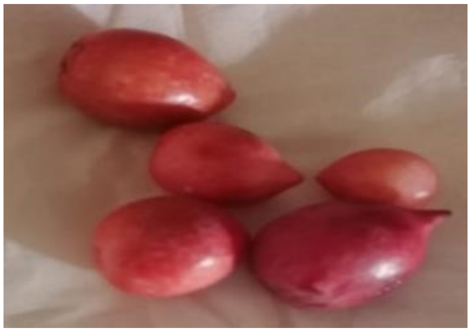

Figure S1. *C. macrocarpa* fruit

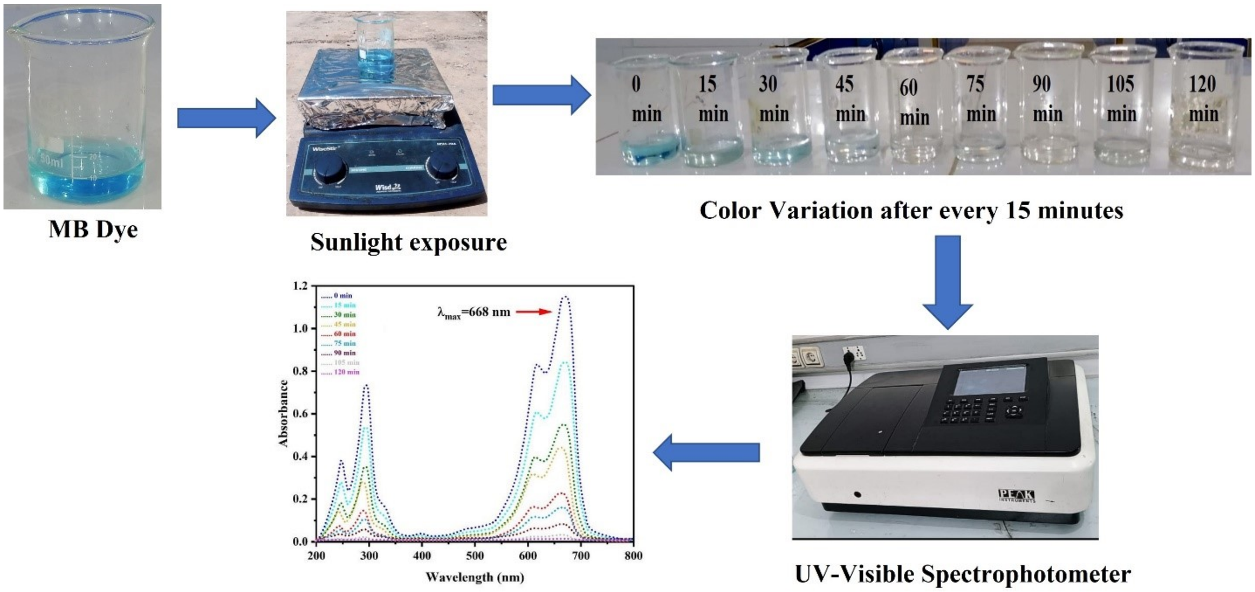

Figure S2. Scheme of photocatalytic activity analysis

Table S1. Details of variables and their coded levels

Independent Variable (unit)	Symbol	Coded levels		Mean
		Low (-1)	High (+1)	
pH	A	3	13	8
Temperature (K)	B	298	358	328
Initial Concentration of pollutants (ppm)	C	10	30	20
Catalyst Dosage (mg)	D	10	50	30

Table S2. Phytochemical screening results for the CMFE extract.

Metabolite	Test	Observation	Result
**Phenols**	Lead acetate test	White precipitates	Positive
**Carbohydrates**	Molisch test	violet ring	Positive
**Tannins**	Braymer's Reagent test	greenish black color	Positive
**Flavonoids**	Shinoda test	pink scarlet color	Positive
**Terpenoids**	Salkowski test	pink scarlet color	Positive
**Saponins**	Foam test	Stable foam	Positive
**Anthraquinones**	Borntrager test	pink color	Positive

**Alkaloids**	Draggendorff test	Brown color	Positive

S1. Calculation of crystal parameters by XRD spectrum

The Debye-Scherrer relation (**Equation a**), the dislocation density (δ) formula (**Equation b**), the micro strain formula (**Equation c**) and degree of crystallinity (**Equation d**) were used to compute the crystal parameters, which include crystallite size, dislocation density, micro strain and degree of crystallinity, respectively [30]. The values of these parameters are depicted in **Table 2**.

$$D = \frac{k\lambda}{\beta \mathrm{Cos}\,\theta} \qquad (a)$$

$$\delta = \frac{1}{D^2} \qquad (b)$$

$$\varepsilon = \frac{\beta}{4\tan\theta} \qquad (c)$$

$$Degree\,of\,Crystallinity = \frac{Area\,of\,crystalline\,peaks}{Area\,of\,all\,peaks} \times 100 \qquad (d)$$

Where 'D' is the crystallite size, 'K' is Scherer constant, 'λ' wavelength of X-rays, 'θ' is the diffraction angle and 'β' refers to the correspond to the FWHM.

Table S3. Fit summary of model by software for MB degradation

Source	Sequential p-value	Lack of Fit p-value	Adjusted R^2	Remarks
Linear	0.4544	0.0008	-0.0077	
2FI	0.9986	0.0004	-0.3148	

**Quadratic**	**< 0.0001**	**0.8471**	**0.9856**	**Suggested**
Cubic	0.7013	0.7649	0.9824	**Aliased**

S2. Butler-Ginley Equations

$$E_{CB} = X - E_C - 0.5E_g \quad (e)$$
$$E_{VB} = E_{CB} + E_g \quad (f)$$

Here, X, E_C and E_g are the Mulliken's electronegativity, the energy of free electrons (4.5 eV) and energy bandgap.

S3. Proposed mechanism of MB degradation

The suitable mechanism of catalytic degradation of MB is represented by **Equations (g-o)**.

$$CuO + h\upsilon(UV - Vis) \rightarrow CuO(e^-(CB) + h^+(VB)) \quad (g)$$
$$H_2O(ads) + h^+(VB) \rightarrow OH^\bullet(ads) + H^+(ads) \quad (h)$$
$$O_2 + e^-(CB) \rightarrow O_2^{\bullet -}(ads) \quad (i)$$
$$O_2^{\bullet -}(ads) + H^+(ads) \; f \; HOO^\bullet(ads) \quad (j)$$
$$HOO^\bullet(ads) \rightarrow H_2O_2(ads) + O_2 \quad (k)$$
$$H_2O_2(ads) \rightarrow 2OH^\bullet(ads) \quad (l)$$
$$MB + 2OH^\bullet \rightarrow \text{intermediates} \rightarrow CO_2 + H_2O \quad (m)$$
$$MB + h^+(VB) \rightarrow \text{Oxidation Products} \quad (n)$$
$$MB + e^-(CB) \rightarrow \text{Reduction Products} \quad (o)$$

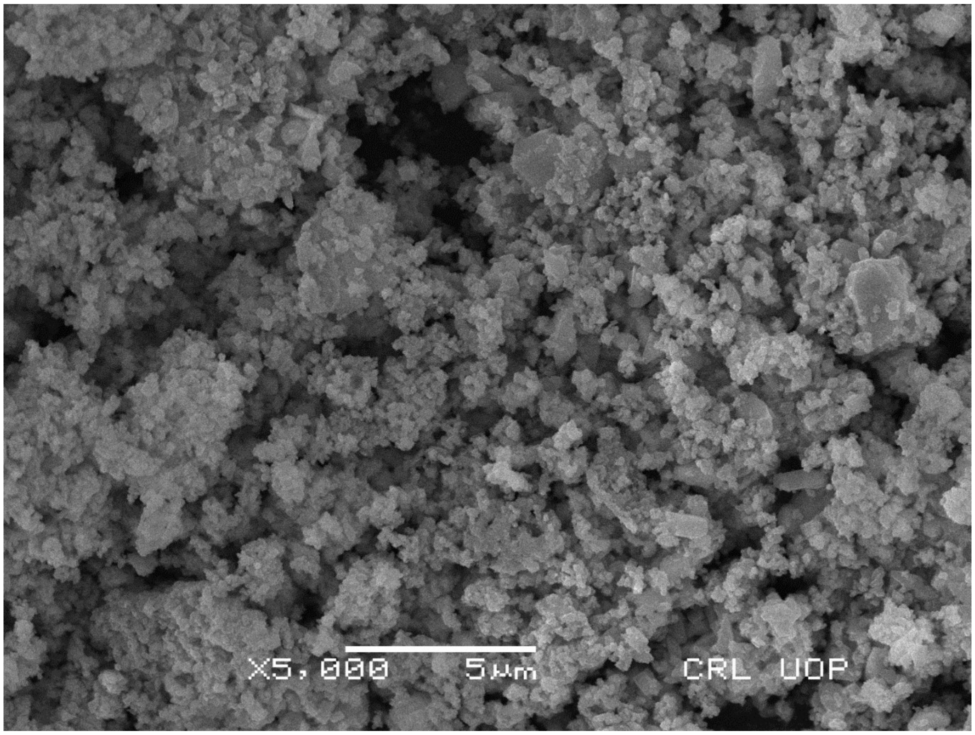

Figure S3. SEM image of CMFE@CuO NPs

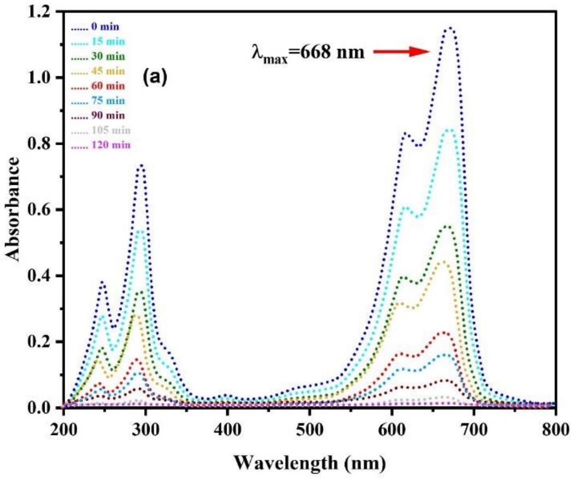

Figure S4. UV-Vis spectra of MB at different time intervals

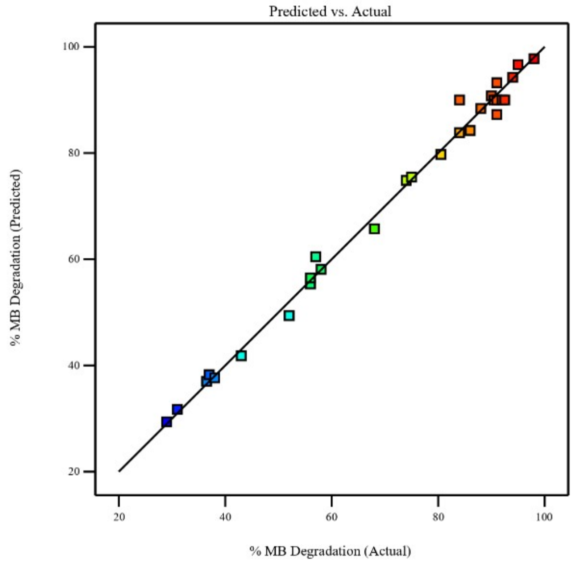

Figure S5. Actual values vs predicted values of MB dye degradation

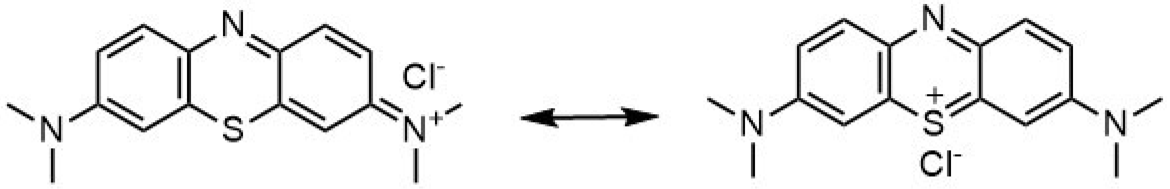

Figure S6. Different resonance forms of MB dye

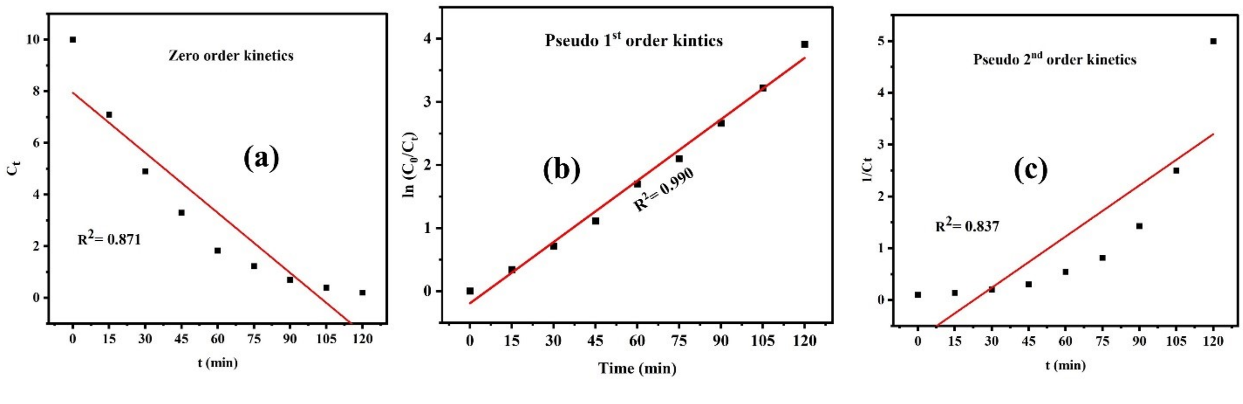

Figure S7. Data fitting of photodegradation of MB on CMFE@CuO NPs (a) zero-order kinetics, (b) Pseudo 1st order kinetics, and (c) Pseudo 2nd order kinetics

Table S4. Comparison table of different catalysts for photodegradation of MB

Catalyst	Degradation (%)	Rate constant (k) (min^{-1})	Reaction time (min)	Light source	Reference
Polythiophene doped ZnO	80	0.0156	180	250 W high-pressure mercury lamp	[1]
CuO NPs	97.5	0.0255	120	UV light	[2]
Cu NPs	90	0.0172	120	sunlight	[3]
MgO NPs	90	0.01544	120	Xenon lamp (> 400 nm)	[4]
$CuO/CuS/MnO_2$ NCs	98	0.022	160	Visible light	[5]
$Zn_{0.5}Cu_{0.5}Fe_2O_4$ NPs	94	0.026	135	UV light	[6]
GO-$LaFeO_3$ NPs	91.2	0.0137	150	Visible light	[7]
CMFE@CuO NPs	99.9	0.044	120	sunlight	Current work

References

1. M. Faisal, F. A. Harraz, M. Jalalah, M. Alsaiari, S. Al-Sayari and M. Al-Assiri, *Materials Today Communications*, 2020, **24**, 101048.
2. A. I. Khedr and M. H. Ali, *Scientific Reports*, 2024, **14**, 29156.
3. S. C. Mali, A. Dhaka, C. K. Githala and R. Trivedi, *Biotechnology Reports*, 2020, **27**, e00518.
4. A. Ahmad, M. Khan, S. Khan, R. Luque, T. Almutairi and A. Karami, *International Journal of Environmental Science and Technology*, 2023, **20**, 1451-1462.
5. M. Abdullah, P. John, M. N. Ashiq, S. Manzoor, M. I. Ghori, M. U. Nisa, A. G. Abid, K. Y. Butt and S. Ahmed, *Nanotechnology for Environmental Engineering*, 2023, **8**, 63-73.
6. O. H. Abuzeyad, A. M. El-Khawaga, H. Tantawy, M. Gobara and M. A. Elsayed, *Journal of Inorganic and Organometallic Polymers and Materials*, 2024, **34**, 2705-2715.
7. M. A. Mutalib, F. Aziz, N. A. Jamaludin, N. Yahya, A. F. Ismail, M. A. Mohamed, M. Z. M. Yusop, W. N. W. Salleh, J. Jaafar and N. Yusof, *Korean Journal of Chemical Engineering*, 2018, **35**, 548-556.
